# Medication overuse headache: position statement of specialized headache centers in Brazil

**DOI:** 10.3389/fpain.2026.1712373

**Published:** 2026-01-27

**Authors:** Abouch Valenty Krymchantowski, Carla Jevoux, Fabiola Dach, Carlos Alberto Bordini, Hilton Mariano Silva Júnior, Pedro Augusto Sampaio Rocha-Filho, Marcelo Moraes Valença, Renata Gomes Londero, Pedro André Kowacs, Élcio Juliato Piovesan, Luiz Paulo Queiroz, Raimundo Pereira Silva-Néto

**Affiliations:** 1 Headache Center of Rio, Rio de Janeiro, Brazil; 2 Universidade de São Paulo in Ribeirão Preto, São Paulo, Brazil; 3 Clínica de Cefaleias de Batatais in Batatais, São Paulo, Brazil; 4 Pontifícia Universidade Católica (PUC) de Campinas, São Paulo, Brazil; 5 Universidade Federal de Pernambuco, Pernambuco, Brazil; 6 Hospital de Clínicas de Porto Alegre, Rio Grande do Sul, Brazil; 7 Universidade Federal do Paraná in Curitiba, Paraná, Brazil; 8 Universidade Federal de Santa Catarina, Santa Catarina, Brazil; 9 Universidade Federal do Delta do Parnaíba, Piauí, Brazil

**Keywords:** medication-overuse headache, primary headache, expert consensus, chronic migraine (CM), treatment

## Abstract

**Objective:**

To develop a position statement based on expert opinions for the management of medication overuse headache (MOH) in Brazil.

**Method:**

This was an observational, prospective, descriptive, and opinion-based study. The experts were in several Brazilian states. Twelve experts who fulfilled the inclusion criteria completed a questionnaire that explored their experiences and approaches to managing MOH in both the private and public sectors.

**Results:**

According to most experts, more than 50% of migraine patients have MOH and psychiatric comorbidities. Experts abruptly stop pain medications, prescribing a bridge treatment for more than 50% of patients. Acute treatment is administered for up to two days per week. Prophylaxis was initiated immediately, and topiramate and monoclonal antibodies were the first choices, respectively, for 36.3% and 54% of professionals. The first follow-up appointment should occur within 4 weeks.

**Conclusions:**

Further guidelines based on evidence as well as expert opinions should be developed for the Brazilian reality, and future prospective studies can be conducted to compare the effects of different treatment regimens for MOH.

## Introduction

Medication overuse headache (MOH) is secondary to the overuse of acute treatments for a primary headache disorder. All classes of drugs used to treat headache attacks may cause MOH, and patients with MOH may have different underlying primary headaches, such as migraine or tension-type headache (1–3).

Medication overuse headache is highly debilitating and costly, affecting 1%–2% of the general population (4–6). There is a female predominance, and the patients may represent the majority of patients seeking help in tertiary centers, with a prevalence as high as 70% among referred sufferers (5–9). Although there is burdensome morbidity with reduced productivity and high absenteeism, limited evidence is available regarding the best treatment strategies (8, 10, 11). The management of MOH is also based on recommendations from expert opinion, despite contradictory scientific evidence. Still, multidisciplinary treatment, high motivation, absence of psychiatric comorbidities, and overuse of drugs other than benzodiazepines, barbiturates, and opioids are favorable outcome factors (8, 11, 12).

However, real-world patients are generally not recruited into the few trials available for migraine or chronic migraine and MOH. Therefore, conclusions from these studies may not apply to real headache patients seeking help in tertiary centers (13, 14). In addition, different geographic realities make this management vary across countries, centers, and even different realities within the same country, like the public and the private sectors in the health system of Brazil (15). These patients are usually considered difficult or even refractory, but most of the time, the lack of a previous broad and thorough approach may have been the real reason for past treatment failures (9, 15). Conversely, tertiary centers frequently provide comprehensive approaches, clearer explanations, and, sometimes, accessory materials for motivating patients to achieve better adherence and outcomes (12, 16, 17). Current available evidence suggests that outpatient detoxification is enough for a successful withdrawal and for the reduction of headache parameters, as well as decreased consumption of overused medications over time (8, 10–12). In addition, sustained response based on advice alone is somewhat difficult, and even among those patients who succeed in detoxification, nearly one-third will relapse within the first year of follow-up (8, 12).

Due to the existing controversies and the absence of a consensus regarding the best treatment strategies based on scientific evidence for MOH, this study was conducted.

## Methods

### Study design

This was an observational, prospective, descriptive, and opinion-based study. The experts who provided their opinions were located in several Brazilian states. Data for this study were collected from June to August 2025.

### Inclusion and exclusion criteria

The experts were defined as clinicians with primary specialization in Neurology, having had at least 15 years devoted to headache patients in tertiary centers, having attended at least 15 headache meetings in Brazil and abroad, and having published at least 10 papers in Headache Medicine.

### Data collection

Twelve experts who met the inclusion criteria completed a questionnaire exploring experiences with MOH management in the private and public sectors. The questions answered by the experts are detailed in Table 1. The two experts from Rio de Janeiro filled out the questionnaires together. The differences between the public and private sectors are meaningful, given the realities of practicing medicine. The public sector is free for everyone and is funded by the government. Most patients are unable to afford the prescribed therapies. Conversely, in the private sector, patients or their health plans pay for care and treatment, making it noteworthy to examine the differences in approaches.

**Table 1:** Experts' position statements on the profile of patients with medication overuse headache.

Position statement	Answers	
	*n*	%
Do you provide services at public tertiary centers?		
No	3	27.3
Yes	8	72.7
Do you provide services at private tertiary centers?		
No	1	9.1
Yes	10	90.9
What is the percentage of your public vs. private patients?		
10% vs. 90%	3	27.3
80% vs. 20%	2	18.2
70% vs. 30%	2	18.2
50% vs. 50%	2	18.2
40% vs. 60%	1	9.1
85% vs. 15%	1	9.1
What is your overall percentage of patients with medication overuse headache?		
20%	1	9.1
30%	2	18.2
35%	1	9.1
40%	1	9.1
50%	2	18.2
75%	1	9.1
80%	3	27.3
What is the percentage of patients with MOH who have a clear history of episodic migraine?		
25%	1	9.1
35%	1	9.1
50%	1	9.1
70%	2	18.2
80%	1	9.1
90%	2	18.2
100%	2	18.2
–	1	9.1
What is the percentage of patients with MOH who have a clear history of episodic tension headache?		
0%	3	27.3
2%	1	9.1
5%	1	9.1
8%	1	9.1
10%	4	36.3
30%	1	9.1
Do you immediately initiate prevention of the underlying primary headache?		
No	0	0.0
Yes	11	100.0
On day 1, what is the percentage?		
0%	1	9.1
40%	1	9.1
95%	1	9.1
100%	6	54.5
After the bridge treatment, what is the percentage?		
0%	6	54.5
5%	2	18.2
70%	1	9.1
100%	1	9.1
After the first follow-up appointment, what is the percentage?		
0%	8	72.7
80%	1	9.1
100%	2	18.2
Do you initiate preventive treatment with one drug, what is the percentage?		
0%	2	18.2
10%	1	9.1
50%	2	18.2
60%	1	9.1
70%	1	9.1
80%	2	18.2
85%	1	9.1
99%	1	9.1
Do you initiate preventive treatment with two drugs, what is the percentage?		
1%	1	9.1
10%	2	18.2
15%	1	9.1
20%	1	9.1
25%	1	9.1
30%	3	27.3
75%	1	9.1
98%	1	9.1
Do you initiate preventive treatment with more than two drugs, what is the percentage?		
0%	4	36.3
2%	1	9.1
10%	2	18.2
20%	1	9.1
25%	2	18.2
80%	1	9.1
What is the overall percentage of your MOH patients whose initial preventive treatment is done with anti-CGRP therapies in the public sector?		
0%	8	72.7
1%	2	18.2
What is the overall percentage of your MOH patients whose initial preventive treatment is done with anti-CGRP therapies in the private sector?		
5%	1	9.1
10%	2	18.2
20%	3	27.3
40%	3	27.3
50%	1	9.1
75%	1	9.1
Do you routinely initiate preventive treatment with a combination of traditional pharmacological agents and some anti-CGRP therapy?		
No	5	45.5
Yes	6	54.5
Do you prescribe any acute treatment in the initial treatment of your patients with MOH?		
No	0	0.0
Yes	11	100.0
Is there a clearly stated maximum weekly frequency of use given to the patient?		
No	1	9.1
Yes	10	90.9
What is the maximum frequency of drug use for the crises?		
2 times a week	5	45.5
3 times a week	3	27.3
As many times as necessary	1	9.1
2 times a week for triptanos, 3 times a week for NSAID's, free—chlorpromazine	1	9.1
What is the percentage for 2 days a week?		
2%	1	9.1
20%	1	9.1
30%	2	18.2
60%	1	9.1
80%	1	9.1
90%	1	9.1
100%	3	27.3
What is the percentage for 3 days a week?		
0%	4	36.3
10%	1	9.1
20%	3	27.3
40%	1	9.1
50%	1	9.1
What is the percentage for variable frequency?		
0%	4	36.3
10%	1	9.1
20%	2	18.2
30%	2	18.2
60%	1	9.1
Pure triptans? What is the percentage?		
0%	3	27.3
10%	1	9.1
20%	2	18.2
40%	3	27.3
50%	1	9.1
60%	1	9.1
Pure gepants? What is the percentage?		
0%	11	100.0
Triptans + NSAIDs? What is the percentage?		
0%	2	18.2
10%	1	9.1
20%	1	9.1
30%	2	18.2
40%	2	18.2
100%	3	27.3
Pure NSAIDs? What is the percentage?		
0%	4	36.3
15%	1	9.1
20%	1	9.1
30%	2	18.2
40%	1	9.1
50%	1	9.1
Other option? What is the percentage?		
0%	3	27.3
5%	1	9.1
10%	2	18.2
20%	1	9.1
30%	2	18.2
Other type of combination? What is the percentage?		
0%	8	72.7
NSAID + chlorpromazine	1	9.1
50% NSAID + triptan	1	9.1
Public vs. Private: Same treatment?		
No [What is the difference?]	7	63.7
Triptans in the private sector and dipyrone in the public	1	9.1
Triptans in the private sector and NSAID in the public	2	18.2
“I don't work in the public sector”	4	36.3
Yes	4	36.3
Do you already prescribe gepants?		
No	8	81.8
Yes	2	18.2
What is the percentage of MOH patients for whom you prescribe gepants for acute treatment?		
0%	7	63.7
Less than 10 patients	4	36.3
What is the percentage of MOH patients for whom you prescribe gepants for preventive treatment?		
0%	7	63.7
10%–15%	4	36.3
Regarding when the first follow-up consultation occurs for your MOH patient: what is the percentage in 4 weeks?		
0%	2	18.2
30%	1	9.1
40%	1	9.1
70%	1	9.1
90%	1	9.1
100%	5	45.4
Regarding when is the first follow-up for your MOH patient: what is the percentage in 8 weeks?		
0%	3	27.3
30%	1	9.1
60%	2	18.2
100%	5	45.4
Regarding when is the first follow-up for your MOH patient: what is the percentage in 12 weeks?		
0%	8	72.7
10%	1	9.1
100%	2	18.2
Do you provide and require the completion of a pain diary?		
No	3	27.3
Yes	8	72.7
Do you provide and require the completion of a digital or written pain diary?		
Digital	3	27.3
Written	5	45.4
I do not provide or require completion	3	27.3
What is the percentage of your MOH patients in whom you CHANGE the approach at the first follow-up if there was NO discontinuation of the overused medication, even without tolerability issues, in the public sector?		
0%	6	54.5
10%	1	9.1
20%	1	9.1
100%	2	18.2
What is the percentage of your MOH patients in whom you CHANGE the approach at the first follow-up if there was NO discontinuation of the overused medication, even without tolerability issues, in the private sector?		
0%	3	27.3
10%	1	9.1
20%	2	18.2
50%	1	9.1
100%	3	27.3
How long after do you schedule the second follow-up for your patient?		
1 month	2	18.2
1,5 months	1	9.1
2 months	3	27.3
3 months	4	36.3
4 months	1	9.1
Is the follow-up time the same in the public and private sectors?		
No	8	72.7
Yes	3	27.3
What is the percentage of your MOH patients who, after 2 months, are using monotherapy for prevention?		
0%	2	18.2
<10%	1	9.1
20%	4	36.3
30%	2	18.2
40%	1	9.1
80%	1	9.1
What is the percentage of your MOH patients who, after 2 months, are using a combination of 2 agents for prevention?		
10%	1	9.1
20%	3	27.2
25%	1	9.1
40%	1	9.1
50%	1	9.1
60%	2	18.2
75%	1	9.1
95%	1	9.1
What is the percentage of your MOH patients who, after 2 months, are using a combination of more than 2 agents for prevention?		
0%	1	9.1
5%	1	9.1
10%	3	27.2
25%	2	18.2
30%	1	9.1
40%	2	18.2
70%	1	9.1
What is the percentage of your MOH patients who, after 2 months, are using any anti-CGRP therapy for prevention?		
5%	4	36.3
10%	2	18.2
20%	2	18.2
50%	2	18.2
60%	1	9.1
What is the percentage of your MOH patients who, after 2 months, are using a combination of any anti-CGRP therapy for prevention + 1 traditional pharmacological agent?		
0%	1	9.1
2%	1	9.1
5%	2	18.2
10%	2	18.2
15%	1	9.1
20%	1	9.1
30%	2	18.2
75%	1	9.1
What is the percentage of your MOH patients who, after 2 months, are using monotherapy for acute treatment?		
0%	3	27.2
5%	1	9.1
10%	1	9.1
20%	1	9.1
30%	2	18.2
50%	1	9.1
70%	2	18.2
What is the percentage of your MOH patients who, after 2 months, are using a combination of 2 or more agents for acute treatment?		
20%	1	9.1
25%	1	9.1
30%	1	9.1
50%	2	18.2
60%	1	9.1
70%	1	9.1
80%	1	9.1
95%	1	9.1
100%	2	18.2
What is the percentage of your MOH patients who, after 2 months, are using a combination of anti-CGRP therapy + triptan for acute treatment?		
0%	3	27.2
3%	1	9.1
4%	1	9.1
5%	2	18.2
10%	1	9.1
30%	1	9.1
60%	1	9.1
95%	1	9.1
What is the percentage of your MOH patients who, after 2 months, are using a combination of anti-CGRP therapy + another pharmacological class for acute treatment?		
0%	3	27.2
1%	2	18.2
2%	2	18.2
5%	1	9.1
10%	1	9.1
40%	1	9.1
70%	1	9.1
What is the average time you perceive for a return to the episodic headache pattern and the end of the excessive use of symptomatic medication in your MOH patients?		
15 days	1	9.1
1–1.5 months	3	27.2
2 months	2	18.2
3 months	2	18.2
4 months	1	9.1
5 months	1	9.1
6 months	1	9.1
What is the percentage of your MOH patients who present psychiatric comorbidities at the first consultation in the public sector?		
0%	2	18.2
50%	2	18.2
70%	3	27.2
80%	2	18.2
100%	2	18.2
What is the percentage of your MOH patients who present psychiatric comorbidities at the first consultation in the private sector?		
50%:	2	18.2
60%	1	9.1
70%	2	18.2
80%	2	18.2
90%	1	9.1
100%	3	27.2
What is the percentage of anxiety cases?		
30%	1	9.1
40%	2	18.2
50%	3	27.2
80%	2	18.2
100%		
What is the percentage of depression cases?		
5%	1	9.1
10%	1	9.1
20%	1	9.1
25%	1	9.1
30%	2	18.2
40%	2	18.2
50%	3	27.2
What is the percentage of personality disorder cases?		
5%	2	18.2
8%	1	9.1
10%	5	45.4
20%	1	9.1
30%	1	9.1
35%:	1	9.1
Do you work together with psychologists and/or psychiatrists?		
No	2	18.2
Yes	9	81.2
What is the percentage of your MOH patients who undergo routine adjunctive psychological treatment?		
10%	3	27.2
15%	1	9.1
20%	3	27.2
35%	1	9.1
40%	2	18.2
50%	1	9.1
What is the percentage of your MOH patients who undergo routine physiotherapy treatment?		
0%	2	18.2
5%	1	9.1
10%	3	27.2
20%	4	36.4
60%	1	9.1
What is the percentage of your patients with chronic migraine without MOH?		
0%	1	9.1
2%	1	9.1
3%	1	9.1
5%	1	9.1
10%	1	9.1
20%	1	9.1
30%	2	18.2
40%	2	18.2
70%	1	9.1
What is the percentage of your patients with MOH without chronic migraine?		
0%	4	36.4
5%	2	18.2
8%	1	9.1
10%	4	36.4

MOH, medication overuse headache; CGRP, calcitonin gene-related peptide; NSAIDs, Nonsteroidal anti-inflammatory drugs.

### Statistical analysis

Data were presented as percentages. The percentage is always related to the total number of experts. All collected data were organized in a database. The BioEstat version 5.0 software was used for statistical analysis.

## Results

Six experts replied that more than 50% of their first-time patients have MOH. Only three experts replied that less than 50% of the patients with MOH had a clear history of episodic migraine. Treatment was initiated by abrupt discontinuation in more than 50% of patients by three experts in the public sector and by 10 experts in the private sector. Nerve blocks were performed for more than 50% of patients in the first week by two experts in the public sector and four experts in the private sector.

Bridge treatment is prescribed for more than 50% of patients by six experts in the public sector and by 10 experts in the private sector. Preventive treatment is immediately initiated for more than 50% of MOH patients using one drug, two drugs, and with more than two drugs by six experts, four experts, and two experts, respectively. Nearly 36.3% of the professionals prefer topiramate as the first choice, while 54% of the professionals included ANTI-CGRP monoclonal antibodies (mAbs) among the top three preferred drugs to start treatment. Anti-CGRP therapies were used as part of a combination therapy by 54.5% of experts in the treatment of MOH.

During the occurrence of headache attacks, drugs were given up to two days a week by five experts and three days a week by three experts. Among all patients, the first follow-up consultation should occur within 4 weeks, according to five experts, within 8 weeks for two experts, and 12 weeks for five practitioners. The percentage of MOH patients for whom the approach was changed at the first follow-up, if no discontinuation of the overused medication was successful, was higher than 50% of patients, as reported by two experts in the public sector and four experts in the private sector.

After 2 months, one drug was used for acute treatment in more than 50% of patients, according to the assessment of three experts, and three experts prescribed two or more drugs. Two experts administered anti-CGRP and triptans to more than 50% of patients for acute treatment after 2 months. Psychiatric comorbidities were present in more than 50% of patients coming to eight experts in the public sector and 11 experts in the private sector. About nine experts frequently work with psychiatrists to treat MOH. About four experts replied that 10% of MOH patients had no chronic migraine. The expert position statement is presented in Table 1.

Table 2 shows a comparison between position statements from experts in the public and private sectors.

**Table 2:** Comparison between position statements from experts in the public and private sectors.

Position statement	Public sector^a^		Private sector	
	%; yes/no	Answers (n; %)	%; yes/no	Answers (n; %)
In the last 6 months, what is the overall percentage of first-time patients with MOH?	0.0	2 (18.2)	5.0	1 (9.1)
	30.0	1 (9.1)	20.0	1 (9.1)
	40.0	1 (9.1)	30.0	1 (9.1)
	60.0	1 (9.1)	40.0	2 (18.2)
	70.0	2 (18.2)	70.0	3 (27.3)
	80.0	1 (9.1)	80.0	1 (9.1)
	90.0	1 (9.1)	90.0	2 (18.2)
	95.0	1 (9.1)	–	–
	–	1 (9.1)	–	–
What is the female-to-male ratio for MOH in the last 6 months and among all your patients	10:1	4 (36.3)	10:1	4 (36.3)
	8:2	2 (18.2)	5:1	1 (9.1)
	5:1	1 (9.1)	4:1	5 (45.5)
	3:1	1 (9.1)	3:1	1 (9.1)
	NP	2 (18.2)	–	–
	–	1 (9.1)	–	–
What is the overall percentage of MOH patients in whom you initiate treatment with abrupt discontinuation of the medication overused	0.0	3 (27.3)	0.0	3 (27.3)
	40.0	1 (9.1)	50.0	1 (9.1)
	80.0	1 (9.1)	80.0	2 (18.2)
	95.0	1 (9.1)	90.0	2 (18.2)
	100.0	4 (36.4)	100.0	5 (45.5)
	–	1 (9.1)	–	–
And what is the percentage of those in whom you do not recommend abrupt discontinuation	0.0	5 (45.5)	0.0	4 (36.4)
	5.0	1 (9.1)	10.0	3 (27.3)
	10.0	1 (9.1)	30.0	1 (9.1)
	50.0	1 (9.1)	50.0	1 (9.1)
	60.0	1 (9.1)	80.0	1 (9.1)
	100.0	1 (9.1)	100.0	1 (9.1)
	–	1 (9.1)	–	–
What is the percentage of your MOH patients in whom you perform blockades in the first week to achieve headache relief	0.0	7 (63.6)	0.0	6 (54.5)
	20.0	1 (9.1)	10.0	1 (9.1)
	90.0	1 (9.1)	60.0	2 (18.2)
	95.0	1 (9.1)	90.0	1 (9.1)
	–	1 (9.1)	100.0	1 (9.1)
What is the percentage of MOH patients in whom you initiate the chosen treatment without discontinuing the overused medication	0.0	7 (63.6)	0.0	5 (45.5)
	5.0	1 (9.1)	10.0	4 (36.4)
	10.0	1 (9.1)	100.0	1 (9.1)
	100.0	1 (9.1)	–	–
	–	1 (9.1)	–	–
Do you provide written explanations or printed materials to emphasize discontinuation	No	9 (81.8)	No	7 (63.6)
	Yes	2 (18.2)	Yes	4 (36.4)
What is the percentage of patients in whom you initiate any bridge treatment in the first week	0.0	3 (27.3)	0.0	1 (9.1)
	40.0	1 (9.1)	80.0	2 (18.2)
	100.0	6 (54.5)	90.0	2 (18.2)
	–	1 (9.1)	100.0	6 (54.5)

a There is a professional who does not work in the public sector.

MOH, medication overuse headache; NP non-proportion.

Table 3 presents the bridging and prophylactic treatments, in order of specialist preference. It was found that most specialists prescribed corticosteroids for bridge treatment, and only one chose the use of triptans. Regarding prophylactic treatment, 36.3% of professionals preferred topiramate as their first choice, and 54% included monoclonal antibodies anti-CGRP among their three preferred medications.

**Table 3:** Preference for bridging, abortive, and prophylactic treatment in patients with medication-overuse headache by specialists, in order of preference.

Expert	Preference		
	Bridge treatment	Abortive treatment^a^	Prophylactic treatment
1st expert	Corticosteroid	“Sumatriptan + naproxen, rizatriptan + naproxen, zolmitriptan + naproxen”	Mabs, topiramate, amitriptyline
2nd expert	Prednisone and indomethacin	“Rizatriptan, NSAID, chlorpromazine”	Topiramate, valproate, fremanezumab
3rd expert	Dopaminergic blocker and prednisone for 10 days	“NSAID, triptan, dipyrone”	Valproate, desvenlafaxine, chlorpromazine
4th expert	Prednisone, NSAIDs	“Rizatriptan, sumatriptan, naproxen”	Topiramate, tricyclics, beta-blockers
5th expert	Prednisone, indomethacin, dexamethasone	“Naratriptan, NSAID, dipyrone”	Nortriptlyne, flunarizine, carisoprodol
6th expert	Chlorpromazine, medication change for the acute crisis, limiting the number of days of use, corticosteroid	“NSAID, triptan, anti-emetic”	Amitriptyline, topiramate, mabs
7th expert	Prednisone, naratriptano	“Chlorpromazine, naproxen, eletriptan”	Topiramate, valproate, propranolol
8th expert	Lidocaine blockade, corticosteroid, NSAIDs	“NSAID, triptan, chlorpromazine”	Topiramate, mab, botox
9th expert	Corticosteroid and I don't use others		Mabs, nortriptlyne + flunarizine, amitriptyline
10th expert	Chlorpromazine		Depends of the comorbities of each patient. In the private sector: mabs, venlafaxine, nortriptyline
11th expert	“Colecoxib, corticosteroids (prednisone), anesthetic blockes + corticosteroids”		Fremanexumab or galcanezumab, topiramate, botox

NSAIDs, Nonsteroidal anti-inflammatory drugs; Mabs, monoclonal antibodies.

a Triptans is included in 100% of the answers.

Specialist preferences for bridging, abortive, and prophylactic treatment in patients with medication overuse headache are shown in Table 3.

Each expert provided a brief report of up to 5 lines describing their most common approach when patients receive the diagnosis of MOH during their first consultation (Table 4).

**Table 4:** Most common approach of 10 Brazilian experts during the first consultation of a patient with MOH.

Experts	Expert's approach
1st expert	“Suggest abrupt discontinuation of painkillers, use up to 2 days per week at most. Nerve blocks, mAbs, treat comorbidities, physiotherapy, psychologist, weight control with a nutritionist, regular physical activity (150 min per week), melatonin 3 mg orally at 7 PM as hormone replacement due to light inhibition before sleeping, we can use topiramate, propranolol, or amitriptyline in combination, headache diary.”
2nd expert	“They are instructed to suspend painkillers or use them, at most, 2 times per week. Prescription for bridge treatment with prednisone for 5 days; immediate start of prophylaxis; provided with a headache diary.”
3rd expert	“I explain the problem; stop the painkillers (prescribe some rescue medication, up to 2–3 days per week); transition therapy; start prophylactics or combine them if they were already taking them.”
4th expert	“Discontinue excessive use, explain everything, start bridge treatment for 5–7 days, and begin prevention with anti-CGRP combined with 2–3 traditional agents. Follow-up in 2 months.”
5th expert	“Counseling to reverse excessive medication use; chlorpromazine as bridge therapy; switch the acute medication used excessively to another class, limiting use to 2 days per week; headache diary; start prophylaxis; antiemetics if nausea; guidance for physical exercise; treatment of psychiatric comorbidities if present; treatment of obesity if applicable.”
6th expert	“I advise the proper use of preventive medication; I emphasize the harms of excessive painkiller use and negotiate a limit for its use.”
7th expert	“Guidance on the condition (during the consultation, leaflet, videos); bridge treatment; prophylaxis; abortive option; option for emergency management; referral to psychologist/psychiatrist/physiotherapist/dentist if comorbidities have been identified; guidance on filling out the diary; follow-up in four to six weeks privately; in eight weeks publicly.”
8th expert	“1) Patient education with slides emphasizing the first 10 worst days of detoxification; 2) Start bridge therapy with corticosteroids; 3) Start mAbs; 4) Early use of painkiller combinations during the crisis with repetitions until the pain disappears, with a limit set at 2 times in 7 days per week.”
9th expert	“Explanation about the disease; Explanation about the negative influence of excessive painkiller use; Start prophylaxis with chlorpromazine combined with another class. Start acute treatment with chlorpromazine without a use limit and another class with a use limit, if chlorpromazine fails.”
10th expert	“Diagnosis of excessive caffeine use, discard confounding factors such as systemic diseases that can facilitate migraine: such as TMJ dysfunction of sleep disorders, hypertension, vagal vasodilatory dysautonomia, postural orthostatic tachycardia syndrome, autoimmune diseases (Asia syndrome, lupus, rheumatoid arthritis, gluten intolerance), insulin hypersensitivity, myofascial syndromes, cervicogenic headache, among others.”

mAbs, monoclonal antibodies; CGRP, calcitonin gene-related peptide, TMJ: temporomandibular joint.

## Discussion

The treatment of patients with MOH has generated considerable and ongoing debate (18). Given the high worldwide prevalence of both chronic migraine and MOH, and the enormous disability and suffering associated with both conditions, an evidence-based answer to this real-world question is a major clinical and research priority. This position statement provides a comprehensive overview of the evidence supporting the various treatment strategies used to manage patients with MOH, at least in Brazil. It does not serve as a country's guideline, nor was it implemented by a specific medical society, given the typical political considerations required to participate in these groups.

MOH is a preventable disease. The emphasis should be on educating patients on the importance of appropriate medication administration and the risks, not only of its side effects, but also the potential development of chronic headaches with excessive medication use (19). Several small studies have emphasized the need for MOH patient education to reduce the incidence of MOH (20). About 75% of all the patients discontinued overused medication in one such study (21). Only 8% of patients know that the potential of overusing all types of headache medication, including those readily available over the counter, could lead to the development of MOH. Written explanations or printed materials to emphasize discontinuation were provided by 18.2% of the experts in the public sector and by 36.4% in the private sector.

There is controversial evidence to support the strategy of early discontinuation of acute medications without concomitant use of other preventive measures. In fact, in most instances where patients have a long history of overusing symptomatic medications and near-daily headache, there is a high non-compliance rate with early discontinuation alone (18, 22, 23). In addition, patients may initially develop several days of worsening symptoms such as headache escalation, nausea, vomiting, sleep disturbances, anxiety, and restlessness (24).

Abrupt discontinuation is particularly helpful for patients who have a higher risk of drug toxicity from overused medication than experiencing withdrawal symptoms. This approach is supported by evidence from open-label clinical trials (25). A guideline from the European Federation of Neurological Sciences recommends abrupt discontinuation of overused medications, initiation of prophylactic therapy just before or at the time of discontinuation, and close, regular follow-up visits. Upon initiation, preventive therapies are to be started at low doses when MOH treatment begins, then titrated up over time. The choice of treatment depends on prior medications, patient preferences, the type of primary headache disorder, comorbidities, and the side-effect profile (26).

To date, treatment with beta-blockers, calcium channel blockers, tricyclic antidepressants, and anticonvulsants has all been successfully utilized (27). There is evidence that topiramate is effective in patients with MOH, especially in chronic migraineurs (10, 28). In the PREEMPT trials for chronic migraines, onabotulinumtoxinA was studied as well. A subset of the patients in the trial had MOH, and a subgroup analysis was performed on those patients who reported benefit in reducing headache days, frequency, and severity. However, the studies did not include patients who overused opioids, and less than 70% of the subjects presented medication overuse (29–31). The COMOESTAS project demonstrated that the combination of detoxification and prophylaxis of MOH patients decreased disability, depression, and anxiety as well (32). This group developed an evidence-based treatment protocol, which, at the end of their trial, demonstrated that 66% of MOH patients no longer overused medications and 47% reverted to episodic headaches (11). Not surprisingly, the dropout rate was higher in the outpatient setting.

Continuing overused medication during initial treatment may be particularly beneficial for patients who are reluctant or afraid to stop the medication and are not at immediate risk of toxicity. Using bridge therapies (temporary medicines for 5–7 days) may be advised for patients with severe or frequent headaches and who are more likely to experience escalating headaches while discontinuing the overused medication.

To help minimize withdrawal symptoms and maximize comfort and adherence during this initial phase, several strategies have demonstrated usefulness. The sufferers need to be educated that the free usage of symptomatic medications should not be an option, nor the previous pattern of drug intake. Few non-steroidal anti-inflammatory agents (NSAIDs) showed effectiveness, such as naproxen, indomethacin, and ketorolac (27, 33). Tizanidine is beneficial as an adjunct to NSAID therapy (34). Steroid therapy, in several current studies, has demonstrated minimal effect on MOH withdrawal symptoms, including fewer headache hours or less severity. However, it helps reduce rescue medication use, which may justify its frequent use by most panel experts and reflect its perceived clinical usefulness during the initial phase of withdrawal (35–38). In addition, antiemetics and neuroleptics have proven useful during this phase, including prochlorperazine, diphenhydramine, promethazine, metoclopramide, and chlorpromazine, although the first three agents are not available in Brazil (27). Infusions with dihydroergotamine (DHE) may be necessary in more complex patients but are also unavailable in our reality.

The treatment must be initiated by abrupt discontinuation in more than 50% of patients, according to three experts in the public sector and 10 experts in the private sector. This discrepancy could be interpreted as an ideological approach to this problem. However, we may speculate that the difference is due to a more comprehensive approach, frequently underscored by appropriate materials and support for private-sector paying patients, in contrast to the loose, sometimes careless approach observed in several instances in the public sector. Nerve blocks are performed in more than 50% of patients in the first week, according to two public-sector experts and four private-sector experts. Bridge treatment is prescribed for more than 50% of patients by six experts in the public sector and by 10 experts in the private sector. Preventive treatment is immediately initiated for more than 50% of MOH patients with one drug, two drugs, and more than two drugs by six experts, two experts, and one expert, respectively. About 36.3% of the professionals prefer topiramate as their first choice, whereas 54% of the panel included anti-CGRP monoclonal antibodies among the top three preferred treatment options. These mAbs are to be used as a part of combination therapy by 54.5% of experts in the treatment of MOH.

### Limitations and strengths of the study

A limitation of this study is that the statement is based exclusively on the practices of a limited number of experts, rather than on national guidelines. However, this position statement presents a strength: detailed practices from real experts with robust clinical experience in MOH treatment in Brazil. In addition, it does not rely solely on membership in specific groups for characterization as a headache expert.

## Conclusion

Based on this position statement, it is plausible to propose prospective studies or trials involving a more representative population of health professionals dedicated to the treatment of medication-overuse headache sufferers.

## Recommendation

Further guidelines based on evidence as well as expert opinions should be developed for the Brazilian reality, and future prospective studies can be conducted to compare the effects of different treatment regimens for MOH.

## Data Availability

The raw data supporting the conclusions of this article will be made available by the authors, without undue reservation.
